# Spinal antinociceptive effect of the PnTx4(5-5) peptide is possibly mediated by the NMDA autoreceptors

**DOI:** 10.1590/1678-9199-JVATITD-2023-0103

**Published:** 2024-11-25

**Authors:** Mariana Murta de Abreu, Nancy Scardua Binda, Marcos Paulo Ferreira Corrêa Alves Reis, Danuza Montijo Diniz, Marta do Nascimento Cordeiro, Márcia Helena Borges, Maria Elena de Lima, Fabíola Mara Ribeiro, Marcus Vinícius Gomez, Juliana Figueira da Silva

**Affiliations:** 1 Department of Pharmacy, Federal University of Ouro Preto (UFOP), Ouro Preto, MG, Brazil.; 2 Center of Technology in Molecular Medicine, School of Medicine, Federal University of Minas Gerais (UFMG), Belo Horizonte, MG, Brazil.; 3 Graduate Program in Health Sciences, Institute of Education and Research, Santa Casa de Belo Horizonte, Belo Horizonte, MG, Brazil.; 4 Professor Carlos Diniz Research and Development Center, Ezequiel Dias Foundation(FUNED), Belo Horizonte, MG, Brazil.; 5 Department of Biochemistry and Immunology, Federal University of Minas Gerais (UFMG), Belo Horizonte, MG, Brazil.

**Keywords:** Analgesics, Spinal cord, Spider toxin, PnTx4(5-5), Dizocilpine, NMDA receptors, Glutamate, OS

## Abstract

Background: Medications currently used to treat pain are frequently associated with serious adverse effects and rapid development of tolerance. Thus, there is a need to develop more effective, and safer medicines for the population. Blocking NMDA receptors (NMDAR) has shown to be a promising target for the development of new drugs. That statement is due to NMDAR activation and glutamate release in the spinal cord which affects chronic pain modulation. Therefore, the aim of this study was to evaluate the possible spinal antinociceptive activity of PnTx4(5-5) toxin. The peptide is purified from the venom of the spider *P. nigriventer* and its affinity for NMDAR and sodium channels Nav1.2-1.6 has already been established. Methods: We compared its effect and safety with MK-801 (NMDAR antagonist) and evaluated its influence on glutamate and reactive oxygen species (ROS) levels in CSF. PnTx4(5-5) was administered intrathecally in the Formalin test and co-administered with NMDA in the Spontaneous pain test. After three minutes of observation, mice cerebrospinal fluid was collected to measure glutamate and ROS levels. Results: The spider peptide inhibited nociception as post-treatment in the inflammatory phase of the Formalin test. Furthermore, it inhibited spontaneous nociception induced by NMDA, being more potent and effective than MK-801 in both models tested. A glutamate rise level in the CSF of mice was significantly reduced by the toxin, but ROS increase was not affected. The animals’ motor skills were not affected by the tested doses of NMDAR inhibitors. Conclusion: In conclusion, the results suggest PnTx4(5-5) may mediate its antinociceptive effect in the spinal cord not only by inhibiting postsynaptic receptors but probably also by acting on autoreceptors. This effect does not affect the motricity of mice at the highest dose tested, which suggests that it has therapeutic potential and safety for use as a painkiller.

## Background

Chronic pain has been defined as pain lasting beyond normal tissue healing time, generally taken to be 12 weeks [1]. It has been a few years now, and the Life Sciences have been very interested in finding new molecular targets and therapeutic agents capable of inhibiting or blocking the transmission of painful stimuli and jointly minimizing the appearance of adverse effects [2, 3]. Among these new molecular targets are NMDA (N-methyl-D-aspartate) receptors (NMDAR). In turn, NMDAR antagonists would be candidates for new therapeutic agents [4, 5], and are the subject of this study.

L-glutamate is the main excitatory neurotransmitter of the central nervous system (CNS) - including primary afferents [6]. It is directly involved in signaling nociceptive transmission in the dorsal horn of the spinal cord. In addition to involvement in neurotransmission, it also carries out different functions including synaptogenesis, synaptic plasticity, connection refinement, and cell death [7, 8]. NMDARs are a type of ionotropic glutamate receptor that was discovered in the 1980s. Their discovery occurred when it was demonstrated that the antagonist MK-801 (dizocilpine) prevented the hyperexcitability of nociceptive neurons in the dorsal horn of the spinal cord. Since then, they have been detected in the brain, where they are associated with learning, memory, behavior, and motor coordination processes [9]. 

At the end of the 1970s, studies with ω-conotoxins obtained from the venom of marine snails of the genus *Conus* [10] boosted basic and clinical research into new treatments for chronic pain. After more than two decades of research, the synthetic version of ω-conotoxin MVIIA extracted from *Conus magus* venom, ziconotide, was approved for use in patients with chronic pain refractory to other treatments. Peptides purified from spider toxins have also been widely studied as they have an analgesic effect [11-13]. An example of that is the Phα1β, a toxin purified from *Phoneutria nigriventer* venom [14, 15]. Several studies have demonstrated its antinociceptive effect in its natural and recombinant form [15-17]. The present paper refers to the PnTx4(5-5) toxin, a peptide also purified from *P. nigriventer* venom with antinociceptive effect [18]. The study by De Figueiredo et al. [19] described its biochemical structure, which has a sequence of 47 amino acids and a molecular weight of 5175 Da. Furthermore, the work also demonstrated that PnTx4(5-5) had the ability to inhibit currents generated by NMDA-type channels using “whole-cell voltage-clamp” techniques in cultured rat hippocampal neurons [19]. Innovative evidence has also demonstrated the affinity of PnTx4(5-5) for voltage-sensitive sodium channels (VSSC). The recombinant toxin rPnTx4(5-5) showed the following decreasing order of affinity for mammalian VSSC: Nav1.3 > Nav1.6 > Nav1.5 > Nav1.4 ≥ Nav1.2 [20]. Apparently, the role of these sodium channels in pain mechanisms has been studied since de first decade of the 21^st^ century [21].

Thus, the objective of this study is to evaluate whether the peptide PnTx4(5-5) has an antinociceptive action on CNS. Moreover, we hypothesize an effect of toxin on spinal cords NMDA autoreceptors and extrasynaptic NMDAR, by discussing its outcome on glutamate and ROS release. 

## Methods

### Animals

Male Swiss mice (25-30 g) were used. The mice were housed in plastic cages with free access to water and food and maintained on a 12 h/12 h light-dark cycle (lights on from 7:00 to 19:00). The experiments were performed in accordance with the current guidelines for the care of laboratory animals and the ethical guidelines for investigations of experimental pain in conscious animals [22]. The Ethics Committee of the Federal University of Minas Gerais, CEUA, authorized the studies (Protocol number 347/2012). We followed the guidelines for the Use and Care of Animals for Research issued by the NIH.

### Drugs

PnTx4-(5-5) toxin was isolated from the spider *P. nigriventer* venom by reverse phase high-performance liquid chromatography (HPLC) and anion exchange HPLC, according to De Figueiredo et al. [19]. NMDA, MK-801, Glutamate dehydrogenase type II, NADP^+^, glutamic acid and 2′,7′-dichlorofluorescein diacetate (DCF-DA) (Sigma-Aldrich - St. Louis, MO, USA) were purchased from Sigma Aldrich. Minocycline hydrochloride was obtained from Tocris Bioscience. Morphine and formaldehyde for the formalin preparation were purchased from Cristália. The lyophilized toxin and stock solutions of the drugs were prepared in Phosphate-buffered saline (PBS) in siliconized plastic tubes, maintained at - 18 ºC, and diluted to the desired concentration just before use. Na_2_HPO_4_, KH_2_PO_4,_ and NaCl are the salt content of the PBS solution. All other reagents were of analytical grade.

### Intrathecal injections

The intrathecal (i.t.) injections were performed according to previously described methods [23]. Briefly, a volume of 5 μL/site was administered using a 28-gauge needle connected to a 10-μl Hamilton micro syringe while the animal was lightly restrained to maintain the position of the needle. Puncture of the dura was indicated behaviorally by a slight flick of the tail. Behavioral evaluation was carried out by researchers who were blind to the drug administration.

### Formalin test

Acute neurogenic and persistent inflammatory nociception were evaluated using the Formalin test [24] with minor modifications. Twenty microliters of saline containing 2.5% formalin were injected subcutaneously (s.c.) into the right dorsal hind paw. The mice were immediately placed in a polycarbonate box positioned in front of a mirror for behavior observations. Nociceptive behavior was quantified by counting the time of licking, flinching, and lifting of the injected hind paw. The measurements were taken in two phases: the first phase (neurogenic) was evaluated during the period from zero to five minutes and the second phase (inflammatory) from 15 to 30 minutes after formalin injection. Time rodents spent licking, and raising their paws was recorded in seconds (s), during each phase. The dose bars for the drugs have been shown in the figure for the neurogenic phase to make it possible to ascertain that all the mice started from the same first-phase formalin response conditions. Therefore, the drug’s effect was indeed evaluated only in the inflammatory phase.

 Intrathecal administration of the test agents was performed using a 5 μL of vehicle (PBS), MK-801 (3-100 nmol/site) or PnTx4(5-5) (100-500 pmol/site). The drugs were i.t. administered nine minutes after formalin injection to evaluate the antinociceptive action only in the anti-inflammatory phase.

### NMDA-induced Spontaneous nociception model

The procedure was carried out according to Urca and Raigorodsky [25], and its objective was to confirm the participation of the NMDA receptor in the inhibition of antinociceptive responses induced by the PnTx4(5-5) toxin. Mice were subjected to intrathecal administration of NMDA (3 nmol/site), in addition to MK-801 (3-100 nmol/site), PnTx4(5-5) (10-300 pmol/site), and Minocycline (2 nmol/site) co-administered with NMDA (3 nmol/site) each. The reaction time in “s” of biting the tail or scratching the hips was recorded, these being indicative of nociception. The animals were observed for a period of three minutes.

### Measurements of glutamate levels in cerebrospinal fluid

The mice were subjected to a puncture in the cisterna magna - immediately after halothane euthanasia - to collect cerebrospinal fluid (CSF) after the end of three minutes of observation of the NMDA-induced Spontaneous nociception model. An average of 20 μL of CSF was extracted from animals. Centrifugation occurs at 10,000 ×g for one minute, and 5 μL of the supernatant was analyzed for glutamate content. The objective would be to verify whether the antinociceptive effect of PnTx4(5-5) (300 pmol/site) and MK-801 (100 nmol/site) would involve a reduction of glutamate levels in the animals' spinal cord by blocking NMDAR. Glutamate measurements were performed enzymatically by measuring the increase in fluorescence due to the production of NADPH in the presence of glutamate dehydrogenase and NADP^+^ [26]. To initiate the assay, NADP^+^ (1.0 mM) and glutamate dehydrogenase (50 U) were added to the CSF samples and 10 minutes after the emitted fluorescence was measured [27]. The excitation wavelength was 360 nm, and the emission wavelength was 450 nm. The experiments were performed using an RF-5301PC spectrofluorometer (Shimadzu, Barueri, SP, Brazil).

### Reactive oxygen species content of the CSF

The CSF samples leftovers from the glutamate assay were assessed for ROS measurements. The method was performed using 2′,7′-dichlorofluorescein diacetate (DCF-DA) (Sigma-Aldrich - St. Louis, MO, USA), a fluorescent probe for the assay [28]. Briefly, 2 μL of the CSF supernatant was incubated with 100 μL of 125-μM DCFH-DA stock solution at 37 ºC for 30 minutes and protected from light. The formation of the oxidized fluorescent derivative DCF-DA was monitored at excitation and emission wavelengths of 488 and 525 nm, respectively, in a fluorescent plate reader (PerkinElmer, Waltham, MA, USA). The levels of DCHF-DA in the CSF of the animals were determined as an indicator of peroxide production from the cellular components of the spinal cord because the influx of calcium through the NMDAR contributes to the production of reactive oxygen species.

### Open-field test

The effect of drugs on spontaneous locomotor activity and exploratory behavior was assessed by the Open-field test, as previously reported [29]. The apparatus was an open field for mice (20 x 12 x 20 cm) where motor activity was measured using an activity monitor that uses three infrared light detectors, each located in a photocell. The animals received intrathecal administration of 5 μL of vehicle (PBS), or PnTx4(5-5) (500 pmol/site), or MK-801 (100 nmol/site). They were placed in the open field and evaluated for five minutes, two hours after i.t. drugs injections. The total distance covered in centimeters (cm) was measured to assess horizontal exploration. The time of “rearings” in seconds was the index for vertical exploration and, the number in units (u) of horizontal detachments from the animal's center of gravity was established as the index for “crossing”.

### Rotarod performance test

This test was carried out with the aim of evaluating changes in the animals' motor coordination due to ataxia or the sedative effect of intrathecal administration of the drugs. The procedure was performed as described by Tsuda et al. [30]. Twenty-four hours before the experiment, mice were trained on the rotarod (3.7 cm in diameter, 12 rpm) for two periods of 60 seconds, with a 60-s interval between them. On the day of the experiment, animals were i.t. injected with 5 μL of vehicle, PnTx4(5-5) (500 pmol/site), and MK-801 (100 nmol/site). Each mouse in each group was subjected to the cylinder rotary two hours after intrathecal administration of NMDAR inhibitors. The number of falls and the latency of the 1st fall were recorded for four minutes.

### Statistical analysis

GraphPad Prism™ software was used to analyze data for statistical significance and curve fitting. The results were expressed as mean ± SEM, and the ID50 - 50% inhibitory dose (ID_50_) values are reported as geometric means accompanied by their respective 95% confidence limits. Animal behavior data were analyzed by one-way analysis or two-way analysis of variance (ANOVA) followed by Student-Newman- Keuls or Bonferroni when appropriate. For the *in vitro* experiments, the results were expressed as mean ± SEM, and analyzed by one-way ANOVA followed by Student-Newman-Keuls test. At last, adverse effects were expressed as median ± interquartile ranges. Then, non-parametric analyses were carried out using the Kruskal-Wallis’s test, followed by the Dunn's Multiple Comparison test when appropriate. Probabilities less than 5% (p < 0.05) were considered statistically significant.

## Results

### PnTx4(5-5) has antinociceptive activity in the inflammatory phase of the Formalin test

**Figure 1.**
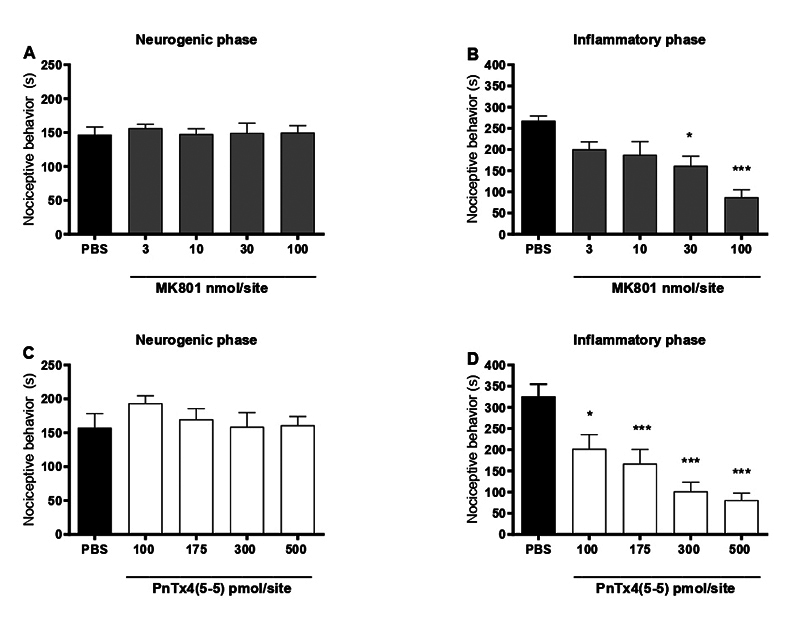
Effect of intrathecal administration of MK-801 and PnTx4(5-5) in the Inflammatory phase of the formalin test. **(A, C)** Control groups of the neurogenic phase of the formalin test in which no drugs were tested. **(B)** Inflammatory phase after administration of MK-801 (3-100 nmol/site). **(D)** Inflammatory phase after administration of PnTx4(5-5) (100-500 pmol/site). Each bar represents the mean ± standard error of 6-8 animals, depending on the group. *p < 0.05, ***p < 0.001 represents the level of significance when compared to animals treated with PBS (one-way ANOVA followed by the Bonferroni test).

Drug-response bar graphs were constructed for PnTx4(5-5) and MK-801 in the Formalin test with the aim of evaluating their possible effects in reversing a previously established nociceptive condition. The intrathecal administration of MK-801 (3-100 nmol/site) nine minutes after formalin injection was able to reduce the inflammatory phase (Figure 1 B), with a calculated ID_50_ of 21.9 (9.28 to 51. 57 nmol/site) and I_max_ of 67.34 ± 6.58%. The calculated ID_50_ for PnTx4(5-5) (100-500 pmol/site) was 104.1 (67 to 161.8 pmol/site) and I_max_ was 76.9± 5.28%. Toxin i.t. administration nine minutes after formalin injection was able to significantly reduce response latency in all tested doses (Figure 1 D ). Bars shown in Figures 1 A  and 1C refer to the response evoked by formalin in the neurogenic phase, for each selected group, prior to drug administration. The mean ± SEM is very close for all groups, as expected for animals that just s.c. formalin.

### PnTx4(5-5) is more potent in a model of spontaneous nociception induced by NMDA than in the Formalin test

**Figure 2.**
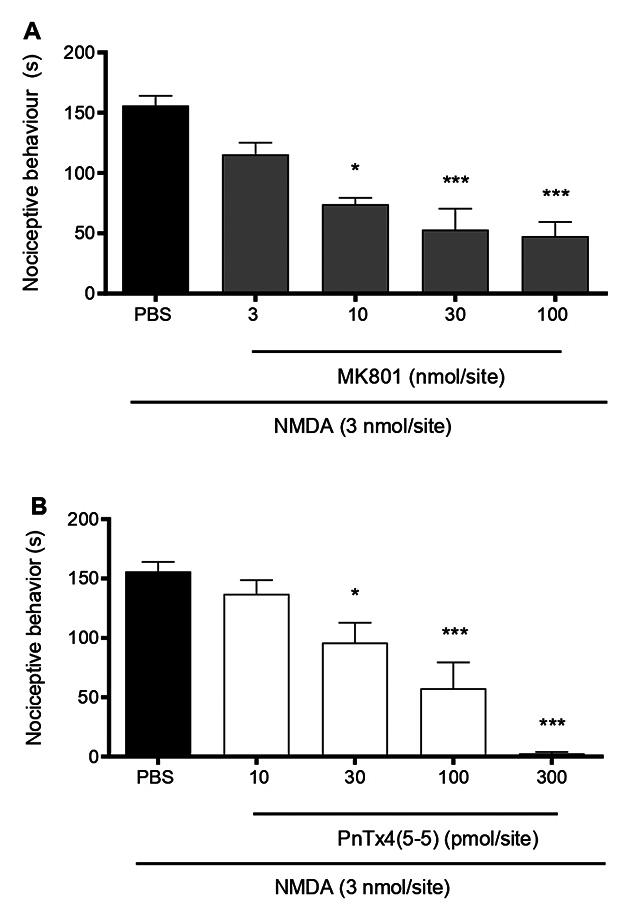
Effect of intrathecal administration of MK-801 and PnTx4(5-5) co-administered with NMDA (3 nmol/site). **(A)** Drug response bar graph of MK-801 (3-100 nmol/site). **(B)** Drug response bar graph of PnTx4(5-5) (10-300 pmol/site). Each bar represents the mean ± standard error of 6-9 animals, depending on the group. *p < 0.05 represents the level of significance when compared to animals treated only with NMDA (3 nmol/5 μL) (one-way ANOVA followed by the Bonferroni test).

Previous studies have demonstrated that PnTx4(5-5) is capable of inhibiting currents evoked by NMDA in hippocampal neurons [19], which corroborates its glutamatergic system-mediated antinociceptive effect in models of peripheral nociception in rats [18]. PnTx4(5-5) has been evaluated in a model of Spontaneous nociception induced by i.t. administration of NMDA because glutamate receptors - specially NMDAR, are important mediators of nociception at the spinal level [31]. The MK-801 (3-100 nmol/site), a blocker of NMDAR, significantly reduced NMDA‐induced nociception at most doses tested (Figure 2 A ). Its calculated ID_50_ was 4.37 (2.4-7.9 nmol/site) and I_max_ was 69.39 ± 7.47%. PnTx4(5-5) (10-300 pmol/site) also significantly reduced nociception (Figure 2 B ), with an ID_50_ of 47.25 (29.77-74.9 pmol/site) and with an I_max_ of 98.2 ± 0.92% (Table 1).

**Table 1.** MK801 and PnTx4(5-5) inhibition indexes on the models of Formalin test and Spontaneous nociception test.

	Formalin test		Spontaneous nociception test	
	MK801	PnTx4(5-5)	MK801	PnTx4(5-5)
	3-100	100-500	3-100	10-300
unit	(nmol/site)	(pmol/site)	(nmol/site)	(pmol/site)
I_max_	67.34 ± 6.58%	7.9 ± 5.28%	69.39 ± 7.47%	98.2 ± 0.92%
ID_50_	21.9 (9.28 - 51.57)	104.1 (67 - 16.8)	4.37 (2.4-7.9)	47.25 (29.77-74.9)

### The antinociceptive effect of PnTx4(5-5) is related only to the reduction of glutamate release in CSF

**Figure 3.**
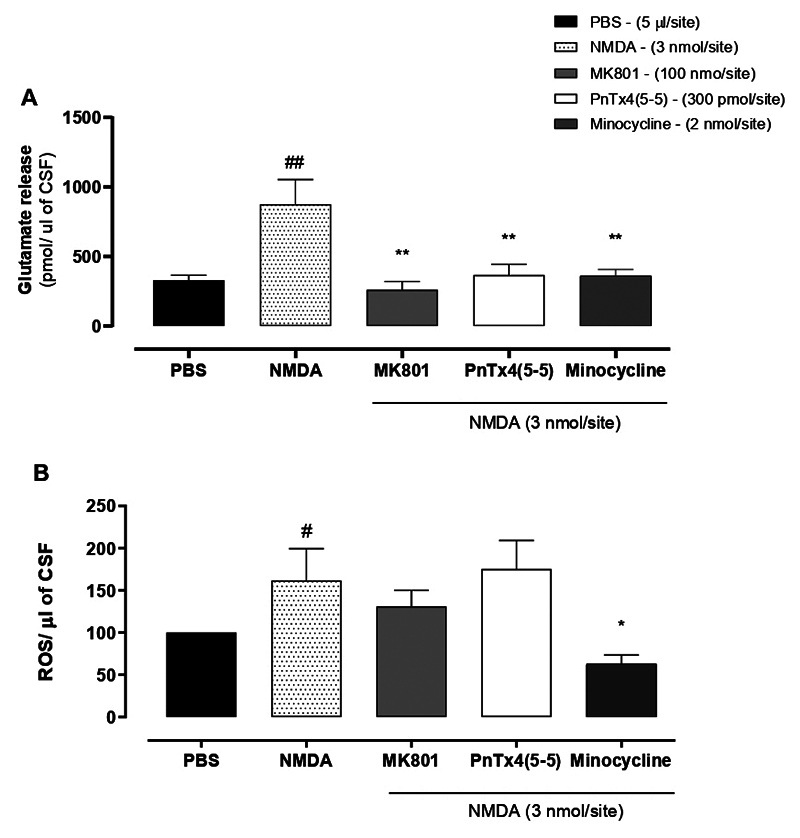
Release of glutamate and ROS in the CSF three minutes after drug intrathecal administration. MK-801, PnTx4(5-5), and Minocycline were co-administered with NMDA (3 nmol/site). **(A)** Measurement of glutamate released in the CSF after treatments. **(B)** Measurement of ROS released in the CSF after treatments - data were normalized in relation to the PBS group. Each bar represents the mean ± standard error of 4-6 animals. #p < 0.05, ##p < 0.01 represents the level of significance when compared to animals treated with PBS. *p < 0.05, **p < 0.01 represents the level of significance when compared to animals treated only with NMDA (one-way ANOVA followed by the Newmann-Keuls test).

The i.t. administration of only NMDA (3 nmol/site) promoted a 166 ± 54% increase in glutamate release when compared to vehicle administration. In turn, co-administration of MK-801 (100 nmol/site) or PnTx4(5-5) (300 pmol/site) was able to significantly reduce the NMDA (3 nmol/site)-induced release of glutamate in the CSF (Figure 3 A ). MK-801 inhibited 70.33 ± 6.76% of the glutamate release induced by NMDA injection, meanwhile, PnTx4(5- 5) inhibited the release of glutamate in 58.15 ± 8.86%. There was no statistical difference when comparing glutamate levels among the MK-801, the PnTx4(5-5), and the PBS group. This result indicates the possible inhibition of NMDA autoreceptors located in the presynaptic terminals of the primary afferents [32]. In addition, it was also investigated whether the reduction in the nociceptive behavior of mice treated with MK-801 and PnTx4(5-5), in the NMDA nociception model, would be related to the decrease in the release of ROS in the CSF. The i.t. administration of NMDA (3 nmol/site) resulted in a 114 ± 44% increase in ROS levels in the CSF, data normalized in relation to PBS. Both MK-801 (100 nmol/site) and PnTX4(5-5) (300 pmol/site) were not able to significantly inhibit the release of ROS in the NMDA nociception model (Figure 3 B ). Minocycline is an antibiotic from the tetracycline class known to be a potent inhibitor of microglia [33]. Minocycline (2 nmol/site) was used as a positive control because microglia are an important source of glutamate and reactive oxygen species [34, 35]. The tetracycline antibiotic inhibited 60.82 ± 8.25% of glutamate release, as well as inhibited 61.19± 7.51% of ROS production.

### PnTx4(5-5) does not affect Open field tests in higher antinociceptive dose

**Figure 4.**
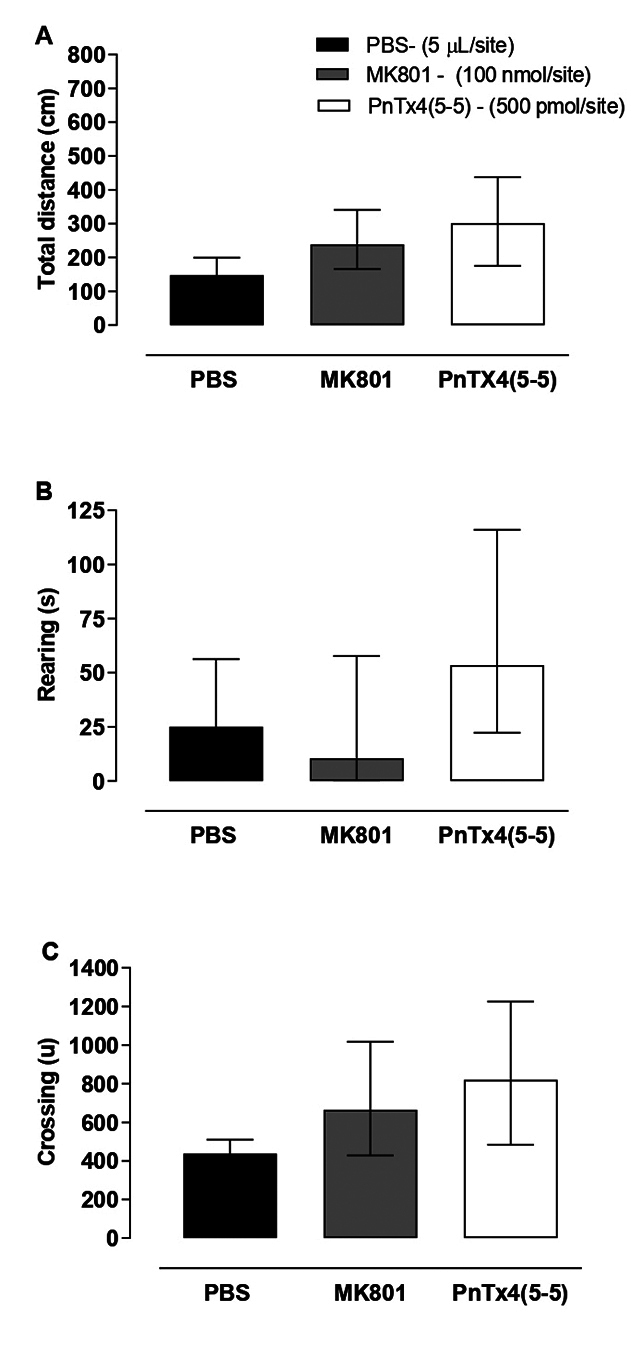
Assessment of exploratory activity two hours after administration of PBS (5 μL/site), MK-801 (100 nmol/site), and PnTx4(5-5) (500 pmol/site). **(A)** Distance covered, **(B)** “Rearing” and **(C)** “Crossing”. Each group represents the mean ± standard error of 6-8 animals. **(B)** Each group represents the median ± 75% interquartile range of 6-8 animals. There was no significant difference between the groups in any of the parameters evaluated (**(A)** and **(C)** one-way ANOVA followed by the Newmann-Keuls test, and **(B)** Kruskal-Wallis’s test followed by Dunn's multiple comparison).

The parameters evaluated were “Rearing” in seconds, the total distance covered in centimeters (cm), and “Crossing” in units. The animals were evaluated two hours after receiving intrathecal administration of MK-801 (100 nmol/site) and PnTx4(5-5) (500 pmol/site). The time of motor activity assessment was chosen based on the peak of Phα1β action in the Hot plate test (SOUZA et al., 2008), and on the motor coordination study with MK-801 performed by Carter [36]. None of the NMDAR inhibitors induced changes in the distance traveled (Figure 4 A), in the “rearing” (Figure 4 B ), or in the “Crossing” (Figure 4 C ).

### Forced locomotor activity is not affected by the highest antinociceptive dose of PnTx4(5-5)

**Figure 5.**
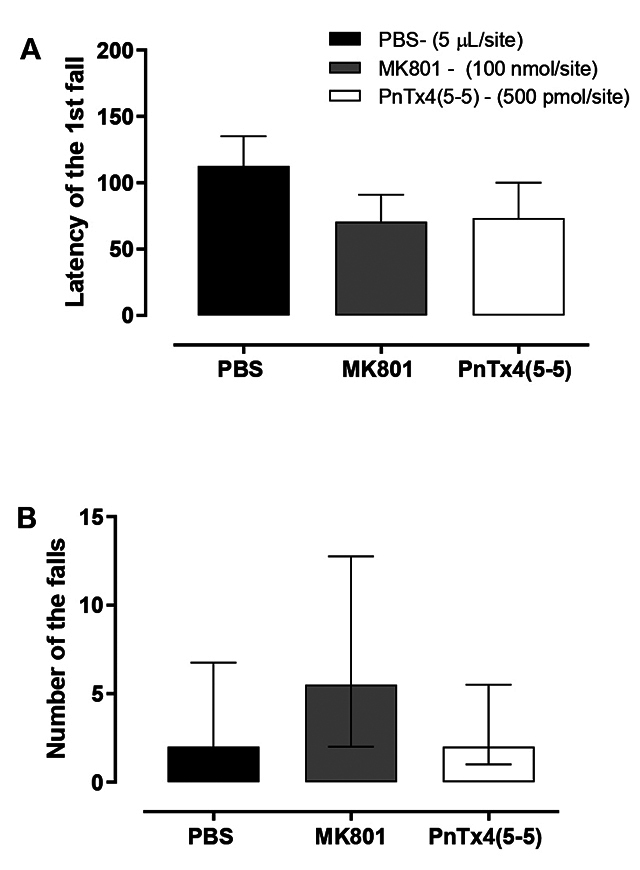
Assessment of forced locomotor activity in a rotating cylinder two hours after administration of PBS (5 μL/site), MK-801 (100 nmol/site), and PnTx4(5-5) (500 pmol/site). **(A)** Latency of the first fall. Each group represents the mean ± standard error of 6-8 animals. **(B)** Number of falls. Each group represents the median ± 75% interquartile range of 6-8 animals. There was no significant difference between the groups. **(A)** One-way ANOVA followed by the Newmann-Keuls test and **(B)** Kruskal-Wallis’s test followed by Dunn's multiple comparison).

The evaluation of forced locomotor activity in a rotating cylinder was carried out using the parameters latency of the 1st fall and number of falls. It is possible to observe that the doses of MK-801 (100 nmol/site) and of PnTx4(5-5) (500 pmol/site) did not cause sedation or significant reduction in motor performance in the observed time range for the latency of the 1^st^ fall (Figure 5 A ) neither for the number of the falls (Figure 5 B ). The same doses were used at the same intervals as in the open-field test.

## Discussion

The venom of the armed spider has toxins with proven antinociceptive potential in different painful modalities [16, 37-41]. Thus, it is a source of pharmacological tools and drugs with potential for clinical use. According to De Figueiredo et al. [19], the toxin PnTx4(5-5) inhibits currents generated by NMDARs, which participate in nociceptive neurotransmission from peripheral structures to higher brain centers [42, 43]. Pharmacological and molecular studies indicate that these ion channels play an important role in controlling nociceptive processes in the spinal cord and contribute to the phenomenon of central sensitization in certain types of neuropathic pain [44, 45]. 

The study began using the model of nociception induced by intraplantar injection of formalin, which is one of the most used tools in screening new compounds with analgesic potential [46, 47]. The Formalin test is characterized by producing a biphasic behavior with a transition from the neurogenic phase - first phase - to the inflammatory phase - second phase - or a state of persistent nociception. There is evidence of the involvement of NMDARs in the development of central sensitization. Its activation is associated with the maintenance of nociceptive impulses in animal models of inflammatory pain, particularly in the second phase of the formalin test [31]. Then, formalin was administered and after nine minutes, PBS, PnTx4(5-5), and MK-801 were i.t. injected. MK-801 was used as a control for blocking the NMDA receptor [48]. It is part of the first generation of NMDA receptor antagonists and was developed in the early 1980s [43].

The objective of this first experiment was to verify the effect of PnTx4(5-5) on a model of nociception involving the NMDA receptor and to verify if this effect would be dose-dependent. As seen in Figures 1 B  and 1D, both the toxin and MK-801 show antinociceptive effects on the inflammatory phase of the Formalin test. Drugs were administered nine minutes after the subcutaneous injection of formalin due to the possibility of carrying out a first screening, exclusively of the second phase of the test. This phase has greater clinical reproducibility since medication is used to reverse a previously set pain condition. According to the literature, MK-801 is a non-competitive NMDAR inhibitor that has an effect only in the inflammatory phase of the Formalin test. Its effect occurs both in pre-treatment [49-51] and in post-treatment. PnTx4(5-5) also had an effect in the second phase of the aforementioned model, promoting evidence that its antinociceptive effect could be credited to a possible blockade of NMDAR.

Next, the effect of the toxin was tested in a model of nociception induced by intrathecal administration of the NMDA pore blocker. The aim of this second experiment was to confirm the hypothesis that the antinociceptive effect occurred through the inhibition of spinal cord NMDA receptors. MK-801, as well as PnTx4(5-5), were co-administered with NMDA. Figures 2 A and 2B confirmed that the antinociceptive effect of the drugs was mediated by NMDAR blockade. As seen in Table 1, PnTx4(5-5) not only inhibited nociception induced by the NMDA but was also more effective and more potent in this model compared to the Formalin test. This can be explained by the fact that the inflammatory phase of the test depends on the participation of other targets, receptors, and ion channels, not only of NMDAR [46, 47]. Besides, PnTx4(5-5) was more potent and effective than MK-801 in both the Spontaneous nociception model and the Formalin test. This suggests that the toxin may have affinity for other targets [20], or for the NMDAR channel in its activated state, just like Memantine - an extrasynaptic NMDAR pore blocker. 

Pain-related synaptic plasticity in the spinal cord is mediated by the activation of postsynaptic NMDA receptors under physiological conditions [52], but this phenomenon is also subject to the influence of presynaptic NMDARs. These receptors once activated contribute to the influx of extracellular Ca^2+^ into the presynaptic terminal, which further stimulates exocytosis. Then, there is an increase in the release of glutamate and substance P in the synapses of the dorsal horn of the spinal cord. We evaluated whether i.t. administration of PnTx4(5-5) would be able to reduce the increase in CSF glutamate levels. The PnTx4(5-5) and MK-801 significantly reduced the increase in glutamate release evoked by spinal administration of NMDA into the CSF. This result (Figure 3 A ) raises the hypothesis the antinociceptive effect of the drugs would possibly be mediated by blocking presynaptic NMDAR and reducing the levels of the receptor's endogenous agonist.

The literature has also demonstrated that the artificial elevation of ROS in the spinal cord induces pain-related behaviors in mice without nerve and inflammatory damage [53-56]. There is also an increase in the production of ROS when NMDAR is activated in central sensitization. These reactive oxygen species promote changes in the phosphorylation of AMPA receptors, which contributes to central sensitization, and painful behaviors. Lee et al. [57] suggest that the reduction of these species would be able to prevent the molecular changes in AMPA receptors and alleviate pain. Therefore, we investigated whether there would be an increase in ROS levels in the CSF of animals after induction of spontaneous nociception by NMDA. We also assessed whether the coadministration of MK-801 and PnTx4(5-5) would be able to prevent its increase. There was a significant increase in ROS in the CSF of animals that received i.t. administration of NMDA compared to PBS. However, MK-801 and PnTx4(5-5) were not able to inhibit the release of ROS induced by NMDA. The hypothesis that MK-801 would have an antioxidant action like Minocycline was based on the proven effect of its metabolites in electron transference [58]. CSF was collected three minutes after the drug’s co-administration, an allegedly very short period to affect ROS’s production pathways. In the present manuscript, authors also presume the quick effect of Minocycline is due to the inhibition of microglia - which contributes to 60% of ROS fast-releasing induced by NMDA. PnTx4(5-5) binds sodium channels Nav1.6 and NMDAR expressed in spinal cord cell membranes. However, it seems necessary to wait longer to collect CSF and verify how channel inhibition by toxins affects ROS production.

Intraperitoneal and intrathecal administration of NMDA receptor antagonists such as MK-801 can cause hyperactivity, hyperreactivity, and sensorimotor deficits [59, 60]. MK-801 is not indicated for clinical use as its adverse effects occur in therapeutic doses and interfere with the animal's physical integrity. Side effects were evaluated using the “Versamax” and “Rotarod” devices, respectively. PnTx4(5-5) (500 pmol/site) and MK-801 (100 nmol/site) did not present side effects such as changes in spontaneous locomotor activity or motor incoordination (Figures 4 and 5). The absence of motor side effects is consistent with the findings of De Figueredo et al. [19], in which mice that received an intracerebroventricularly injection of 30 µg of PnTx4(5-5) - equivalent to 58 nmol/site) did not show any indicative sign of visible toxicity. The administration time was chosen according to the results of the studies of Carter and De Souza et al. [36, 37]. It was expected to observe motor deficits in animals subjected to i.t. administration of MK-801 within two hours. Carter et al. [36] observed that such motor impairments tended to disappear 120 minutes after administration of the non-selective NMDAR antagonist. The motor deficit would probably have been detected if the tests were performed at intervals of 30 and/or 60 minutes. 

According to D’Mello and Dickenson [61], glutamate released by primary afferent fibers in the spinal cord acts on AMPA and NMDA receptors, respectively. Given the high and persistent stimulation of C fibers, there is amplification and prolongation of the responses of neurons located in the dorsal horn of the spinal cord. So, this increase in activity is the result of the activation of NMDA-type glutamatergic receptors. However, when there is only a low frequency of noxious or tactile stimuli, there is no possibility of NMDAR activation. This occurs in acute pain - the first phase of the Formalin test or hot plate test, for example. In this condition, the NMDAR ion channel is blocked by physiological levels of Mg^2+^. This ionotropic receptor requires membrane depolarization, thus allowing the activation of NMDARs to occur and consequently the opening of the calcium channel. Without a doubt, the most intriguing results of this study concern the relationship between the data in Figure 3 and the phenomena of central sensitization and oxidative stress. Neurons and glia are the source of the glutamate and ROS found in the CSF. NMDA, under the described conditions, stimulated an increase in both glutamate and ROS levels in a short period of time. Then, NMDAR was the target that triggered those outcomes, and we could suggest that both MK-801 and PnTx4 (5-5) possibly blocked neuronal NMDA autoreceptors due to the similar result found in glutamate and ROS content. Note that the inhibition values presented in the results of this manuscript are quantitatively very close. Still following this line of reasoning, the influence of Nav1.2-1.6 on the PnTx4(5-5) outcomes may be minimal. After all, the higher decline in glutamate level was caused by MK-801, which, as we know, does not bind to sodium channels. The glial role has been widely described in the literature [62, 63]. It is known that astrocytes, as well as microglia, can contribute to the release of glutamate and ROS, and to the control of pre and post-synaptic activity [62-64]. However, those cells’ contribution to neuroinflammation takes time [64]. and as neither MK-801 nor PnTx4(5-5) affected ROS content, further research is necessary to clarify) antinociceptive mechanisms of PnTx4(5-5). Therefore, in the present study, it was found that the spinal antinociceptive activity of PnTx4(5-5) coincides with states of central sensitization, in which there is a recognized participation of NMDAR and VSSC from neurons as well as from glia [31, 65-68]. 

## Conclusion

MK-801 and PnTx4(5-5) toxin showed an antinociceptive effect in nociception models due to inhibition of NMDAR. This receptor is activated in cases of central sensitization in the spinal cord in which there is pain of difficult treatment. The activation of NMDAR makes pain management challenging with the drugs currently available. Therefore, the search for more effective painkillers continues [69, 70]. PnTx4(5-5) inhibits both the NMDAR and the VSSC, probably contributing to some extent to its analgesic effects. PnTx4(5-5) did not present adverse motor effects at the highest therapeutic dose tested. Mice did not exhibit any adverse motor effect after receiving the highest therapeutic dose of PnTx4(5-5). Thus, the spider peptide becomes a candidate as a new drug for the treatment of persistent and difficult-to-treat chronic pain. At last, we highlight the need for further studies to investigate in depth the mechanisms related to the analgesic effects of the peptide on the CNS, as well as its effectiveness in clinically relevant pain models.

### Abbreviations

cm: centimeters; CNS: central nervous system; CSF: cerebrospinal fluid; DCF-DA: 2′,7′-dichlorofluorescein diacetate; HPLC: high performance liquid chromatography; i.t.: intrathecal; ID_50_: 50% inhibitory dose; I_max_: maximum inhibition: NMDAR: N-methyl-D-aspartate receptors; PBS: phosphate-buffered saline; ROS: reactive oxygen species; s: seconds; s.c.: subcutaneous; VSSC: voltage-sensitive sodium channels.

## Availability of data and materials

 The datasets generated during and/or analyzed during the current study are available from the corresponding author upon reasonable request.
